# Using an Extracurricular Honors Program to Engage Future Physicians Into Scientific Research in Early Stages of Medical Training

**DOI:** 10.1007/s40670-018-0565-y

**Published:** 2018-04-12

**Authors:** Belinda W. C. Ommering, Peter J. van den Elsen, Jolanda van der Zee, Carolina R. Jost, Friedo W. Dekker

**Affiliations:** 1 Center for Innovation in Medical Education, Leiden University Medical Center, Zone V7-P, PO Box 9600, 2300 RC Leiden, the Netherlands; 2 Department of Immunohematology and Blood Transfusion, Leiden University Medical Center, Leiden, the Netherlands; 3 Department of Molecular Cell Biology, Leiden University Medical Center, Leiden, the Netherlands; 4 Department of Clinical Epidemiology, Leiden University Medical Center, Leiden, the Netherlands

**Keywords:** Honors, Extracurricular research program, Physician-scientists, Scholarly concentration

## Abstract

Physician-scientists are urgently needed to make progress in the dynamic world of medical healthcare. Currently, there is a worldwide shortage in physicians pursuing a scientific career. Actively engaging students in research in early stages of medical training could help to direct students towards a scientific career and contribute to creating the next generation of physician-scientists. Leiden University Medical Center (LUMC) implemented an extracurricular Honors program with a fundamental orientation towards research. The program starts in the second year of medical training and is comprised of four different tracks in order to attract multiple types of students with different interests. All four tracks offer students scholarly experiences, but differ in content and amount of provided structure. The LUMC Honors program has a clear goal to develop future physician-scientists, and combined with its unique multiple-track model, the program accommodates about 70 students (25%) each year. The number of students in the program has grown and students’ experiences are positive.

## Background

The Canadian Medical Education Directives for Specialists (CanMEDS) distinguishes being able to use and being able to conduct research as two core competencies of a scholar [1]. A common belief in the medical field is that all physicians should be able to use research, which is important in forming evidence-based decisions and making a grounded diagnosis [2–5]. In order to integrate scientific knowledge in clinical decisions and to ensure that patients receive the best possible healthcare, physicians should be aware of the latest developments in medicine. Being able to critically appraise scientific literature is key in the process of using research in daily clinical practice [6].

However, besides all physicians using research, physicians who actually conduct research are needed as well. These physicians are needed because it is important to create new knowledge to make progress in the demanding world of medical healthcare [3, 4, 6, 7]. Physicians combining clinical work with doing research in the medical context are called physician-scientists. Physician-scientists offer an opportunity to bridge the gap between science and clinical practice [8–10]. They have the opportunity to identify clinical problems in daily practice, which can be translated into research questions and designs [11]. Subsequently, physician-scientists can translate research outcomes into clinical practice [12].

Currently, there is a global shortage in the number of physician-scientists, with too few physicians pursuing a scientific career [2, 8, 10, 13, 14]. A decline in interest for research among physicians in Canada, the USA, and Europe has been documented [2]. How physicians can be directed towards a scientific career is still a topic of debate, although early engagement of medical students in research is mentioned as a possible solution [2, 6, 7, 15–18]. Engaging students in research in early stages of medical education could help to identify a possible scientific career path for these future physicians, as it could trigger enthusiasm and motivation for doing research [4]. This view is shared internationally, as is reflected in the growing amount of curricular and extracurricular courses to engage students in research [2, 19].

Some medical schools have designed and implemented mandatory courses in the curriculum with the goal to get students acquainted with research, for instance Duke University implemented a mandatory period of research into the third year of the medical curriculum, and Stanford University integrated mandatory research experiences for medical students through all years of medical training [19, 20]. Leiden University Medical Center (LUMC) implemented a curriculum change in 2012, integrating mandatory research courses in undergraduate medical training with the purpose to engage students in research in early phases of medical training by providing them with active learning experiences [21].

Besides mandatory courses in the curriculum, a trend is evolving in which medical schools design and implement extracurricular research programs with the aim to involve students in research. These research-based programs occur in very different ways and under different names, but share a mutual goal to expose students to in-depth inquiry and research experiences with capstone projects like writing a thesis or a publishable article. Such extracurricular research programs occur as, for instance, MD/PhD programs, scholarly concentration programs, Capstone programs, Summer Research programs, and Honors programs [19, 22, 23]. Leiden University Medical Center designed and implemented an extracurricular research-based Honors program to engage students in research. The aim of this monograph is to provide a detailed description of the LUMC Honors program to act as reference or inspiration for other medical schools exploring options to implement an extracurricular research-based program.

## The Medical Honors Program of the LUMC

As a faculty that is part of a research intensive university, the LUMC emphasizes the importance of research and educating future physician-scientists. Therefore, one of the goals of medical training is to stimulate students to become familiar with and engage in research. Students of the LUMC follow mandatory courses throughout all years of medical training, in which the students are actively engaged in doing small-scale research projects [21, 24, 25]. Next to these mandatory courses, the LUMC offers an extracurricular Honors program for bachelor students with a fundamental orientation towards research.

The medical Honors program of the LUMC differs from traditional Honors programs in its design and main goal, as the program originated from a point of view that future physician-scientists are needed. Hence, the program is largely devoted to make the first step to cultivate the next generation of physician-scientists. The LUMC program can be seen as an extracurricular research program, internationally similar to earlier mentioned ‘scholarly concentration programs’ [22]. The LUMC Honors program has a relatively large capacity to accommodate students and focuses on providing them with scholarly experiences. It is an extracurricular two-year program and it has a minimum of 30 ECTS (ECTS = European Credit Transfer and Accumulation System), which means that students have to invest 30 × 28 h of active study. The program gives students the opportunity to experience research in an authentic learning situation by actually designing and implementing their own research in one of the departments of the LUMC.

The program starts with an optional orientation phase in the first year of the medical study. All first-year medical students are invited to participate in this orientation phase, which is already unique, as most Honors programs only target so called ‘excellent’ students [26]. The orientation phase consists of multiple expert lectures covering the different (bio)medical research areas of the LUMC. These lectures are given by highly experienced physician-scientists. This allows students to become familiar with and inspired by research and research-related work, and to get to know the career opportunities as future physician-scientists. The number of students who use this opportunity and are actively involved in the orientation phase differs every lecture, ranging from 30 to 150 out of approximately 300 students in total. Students participating in the orientation phase are provided with a possibility to earn some credits for the actual Honors program, prior to the official start in the second year of medical training. Students participating in the orientation phase can choose to submit written reports of the expert lectures (in English), summarizing their content and adding new knowledge from recent literature. The reports are graded by educators specialized in academic writing, scoring students both on content and academic English. If students receive a passing grade for at least four of the meetings, they already earn two credits (2 ECTS) for the Honors program, before the official program has even started. After this, students can choose to earn another credit (1 ECTS) by interviewing one or more post-docs and/or PhD students. Students are asked to make a scientific report on the content and outcomes of the interviews, which again will be graded by educators specialized in academic writing. These interviews provide the students with an even better impression of the ins and outs of performing research.

The orientation phase starts in November and ends in June of the first year of medical study. Students are not obligated to actively participate in the orientation phase; they can still participate in the actual Honors program regardless. By and large, the main goal of the orientation phase is to offer students the possibility to get acquainted with research and the program, and to help students decide if the Honors program seems to be a good fit for them as an extracurricular activity during medical training.

During the summer break between the first and the second year of medical training students need to decide whether they would like to participate in the actual Honors program starting in September. They can officially apply during the summer months. The selection for the program is largely based on self-selection of the student, as three of the four tracks are open to different types of students without having institutional selection criteria. The MD/PhD track is the only track with limited availability, resulting in institutional selection focusing on high grades. Although the selection of the MD/PhD track is comparable to most regular Honors programs, the self-selection of students in the other three tracks is a second factor distinguishing this program from regular Honors programs.

## Four-Track Model of the Honors Program

The Honors program at the LUMC provides four different tracks to attract multiple types of students with different interests. At the start of the program, the student can choose between one of the following four tracks: the MD/PhD track, the Journey into Biomedical Sciences track, the Clinical Research/Epidemiology track, and the Free Research track. The four tracks are different in content and approach, but they share a fundamental orientation towards research. The distribution of students among the four tracks at the start of the program in the years 2013–2016 is illustrated in Table 1.

**Table 1:** Overview of participating Honors students in 2013, 2014, 2015 and 2016

	2013	2014	2015	2016
Medical students	275	265	306	299
Honors students	59	64	72	81
MD/PhD	10	10	10	10
Journey into Biomedical Sciences	13	17	15	18
Clinical Research/Epidemiology	17	15	28	25
Free Research	19	22	19	28

The *MD/PhD track* prepares the student for, and could be the beginning of, a future PhD project. Although every track can be a foundation for a future PhD project, the MD/PhD track explicitly acknowledges this as its main goal. The track has a limited capacity, accommodating only ten students every year. Students are selected by the institution, thus in this regard the track is comparable to regular Honors programs. Students are selected based on academic performance, curriculum vitae, and motivation. The selected students are free to choose a department, and autonomous in choosing their research topic. The coordinator of the Honors program and the supervisor from the department assist the student in making these choices. The department coaches the student in the same way as it would coach a regular PhD candidate. Next to their bachelor studies, students have the opportunity to perform research and to write at least two scientific papers for their future PhD thesis.

The *Journey into Biomedical Sciences track* is a program for students who would like to acquire a deeper understanding of biomedical sciences and it offers an opportunity to follow a program with a fundamental orientation towards biomedical research. Both the first and second year of this track focuses on theory and laboratory skills, and upon completion gives the student the opportunity to combine the Medicine master and the Biomedical Sciences master in Leiden. Main subjects of this track are cellular communication, medical genetics and immunology in the first year, and Molecular Biology and Oncology, communication in science, writing a review, and acquiring and application of laboratory skills in the second year.

The *Clinical Research/Epidemiology track* offers both the opportunity to participate in courses about clinical epidemiology and statistics, and the possibility to do a clinical research project. Students can choose what kind of clinical research they would like to do and are mentored by a senior researcher of the department. An example of a course in this track is ‘Introduction in Clinical Scientific Research’, where students get to know the different departments of the LUMC. This is a popular course and it helps students with the orientation towards a specific department, where they could possibly do their research. Another course of this track gives an introduction in epidemiology by discussing the book of Kenneth Rothman (‘Epidemiology, an introduction’) [27]. The Clinical Research/Epidemiology track also offers an opportunity to follow a 5-day masterclass course in clinical epidemiology, in which students are educated in content identical to the education of PhD candidates and clinical researchers. In both the first and the second year of this track, the student will actively participate in doing research.

The last track is the *Free Research track*, which offers students the possibility to do a research project in a department of their own choice. This track seems suitable for two types of students. First, this track appeals to students who are eager to do research, but who are not yet ready for long-term commitment, as they are still curious to find out which type of research and (bio)medical research area suits them best. Second, this track is also attractive for students who already decided what kind of research they want to do and at what department, committed to doing their own research in one particular area. The Free Research track offers these students the possibility to devote their Honors program to conducting their own research at one department.

In contrast to the MD/PhD track, the other tracks are mostly self-selected. Notably, the Honors program of the LUMC is designed with maximum flexibility for the participating students. If students come up with an individualized plan to earn their 30 credit points, they can approach the Honors coordinator to discuss the possibilities. Additionally to earning credits in any of the four tracks or from the alternative propositions, all students are required to follow at least one Honors Class of 5 ECTS offered by the University of Leiden. The Honors Classes of all Leiden Faculties are coordinated by the Honors Academy Leiden, and are always interdisciplinary by nature. Most of the Honors Classes are in English, and are aimed at broadening students’ intellectual horizon. There is a great variation in the offered Honors Classes and the students are free to choose whether they prefer to follow an Honors Class in their second or third year.

Summing up, to receive an Honors Certificate students need to have earned 30 ECTS, followed an Honors Class, and passed all the courses in the regular curriculum.

## Outcomes of the LUMC Honors Program

The LUMC Honors program is fundamentally orientated towards research and has the clear goal to develop future physician-scientists. The relative large capacity, self-selection, and four different tracks to address different types of students contribute to the uniqueness of the program as compared to other, more traditional, Honors programs. Designing an Honors program in this way seems to be attractive for students, as is reflected in the increasing number of participating students over the last few years. From the program’s inception with about 20 students participating in 2010, nowadays, the program provides extracurricular research activities for 81 medical students in 2016 (Table 1). This number represents 27.1% of one yearly cohort. Most of the extracurricular Honors programs provide only places for few students, so having 27.1% of all students in a year participating in an extracurricular program is truly exceptional.

Students of the LUMC Honors program were surveyed in 2016 to evaluate the program and its four different tracks (*n* = 97). A total of 136 Honors students were approached, of whom 97 students participated (71.3%) and anonymously filled out a digital questionnaire regarding their experiences in the program. Students from both years of the program and all four different tracks were included. The sample consisted out of 22 students from the MD/PhD track, 18 students from the Journey into Biomedical Sciences track, 20 students from the Clinical Research/Epidemiology track, and 30 students from the Free Research track. From the other seven students, data regarding the track in which they participated was missing. The evaluation showed that 92.9% of the students were satisfied with the way the goals and opportunities of the program were communicated. Overall, the evaluation indicated that the Honors program offered by the LUMC is well appreciated by the participating medical students because it allows flexible implementation of the offered tracks and the possibility to propose alternative plans. The four tracks of the program differ regarding their provided structure; therefore, the Honors program as a whole may appeal to different types of students. A tailor made program that suits students’ individual needs and aspirations may be the key to success. We believe this contributes to the large amount of students participating every year. Moreover, most students who follow the LUMC Honors program would recommend the program to their fellow students.

Additionally, students who recently started the Honors program were interviewed (*n* = 6). The students were approached during an Honors meeting and asked if they wanted to participate in orientating interviews. Participation was voluntary and interviews were conducted by the first author. The aim of the interviews was to provide insights into reasons students have to participate in the Honors program. Curiosity in combination with a need for extra challenge seemed to be the most important reason to participate in the program, as one student said: *‘Challenge is the biggest reason and also, I want to discover new things. I want to understand why something works and not only that it works. That is something I miss in my study’.* Another motive is that students want to learn more about doing research because of their interest in a research oriented career, one student stated: *‘I really want to be a researcher. So the sooner [to start with doing research] the better’.* Students also discussed the benefits of doing extracurricular research for possible future choices. One student mentioned research being *‘very good for your resume’* and another student said *‘if you want to be a specialist, it is self-evident to follow a PhD first. I don’t know what kind of specialist I want to be, if I want to specialize at all, but I want to keep my options open. So it seems good that when I do know what I would like to do, I have extra possibilities to do it. That is why it seems good to become familiar with research and that is why I chose the Honors program as extra activity’*. Finally, students mentioned that participating in the Honors program was a *‘perfect way to build a network’*.

## Conclusion

An extracurricular Honors program, aimed at directing more students towards a scientific career as physician-scientists, appears to be an effective tool to actively engage undergraduate students in research. By offering the program to more students than only the obviously highly talented ones, and by providing different tracks of interest, the program clearly reaches more students. The long-term effects of this program still need to be evaluated by analyzing the actual career choices our students make, number and impact of publications, scientific presentations and research-related advanced degrees like a PhD. However, initial experiences of students seem positive already, as is reflected in both the outcomes of the evaluation and the growing number of students participating in the Honors program.
